# Mitochondrial Carriers for Aspartate, Glutamate and Other Amino Acids: A Review

**DOI:** 10.3390/ijms20184456

**Published:** 2019-09-10

**Authors:** Magnus Monné, Angelo Vozza, Francesco Massimo Lasorsa, Vito Porcelli, Ferdinando Palmieri

**Affiliations:** 1 Department of Biosciences, Biotechnologies and Biopharmaceutics, Laboratory of Biochemistry and Molecular Biology, University of Bari Aldo Moro, Via E. Orabona 4, 70125 Bari, Italy; 2 Department of Sciences, University of Basilicata, Via Ateneo Lucano 10, 85100 Potenza, Italy; 3 Center of Excellence in Comparative Genomics, University of Bari, via Orabona 4, 70125 Bari, Italy; 4 CNR Institute of Biomembranes, Bioenergetics and Molecular Biotechnologies (IBIOM), 70126 Bari, Italy

**Keywords:** mitochondrial carrier, mitochondrial transporter, membrane transport, amino acids, aspartate, glutamate, ornithine, arginine, lysine, glycine

## Abstract

Members of the mitochondrial carrier (MC) protein family transport various molecules across the mitochondrial inner membrane to interlink steps of metabolic pathways and biochemical processes that take place in different compartments; i.e., are localized partly inside and outside the mitochondrial matrix. MC substrates consist of metabolites, inorganic anions (such as phosphate and sulfate), nucleotides, cofactors and amino acids. These compounds have been identified by in vitro transport assays based on the uptake of radioactively labeled substrates into liposomes reconstituted with recombinant purified MCs. By using this approach, 18 human, plant and yeast MCs for amino acids have been characterized and shown to transport aspartate, glutamate, ornithine, arginine, lysine, histidine, citrulline and glycine with varying substrate specificities, kinetics, influences of the pH gradient, and capacities for the antiport and uniport mode of transport. Aside from providing amino acids for mitochondrial translation, the transport reactions catalyzed by these MCs are crucial in energy, nitrogen, nucleotide and amino acid metabolism. In this review we dissect the transport properties, phylogeny, regulation and expression levels in different tissues of MCs for amino acids, and summarize the main structural aspects known until now about MCs. The effects of their disease-causing mutations and manipulation of their expression levels in cells are also considered as clues for understanding their physiological functions.


**Contents**

1. Introduction22. Mitochondrial Carriers for Acidic Amino Acids72.1. The Human and Yeast Aspartate-glutamate Carriers72.2. Arabidopsis AtUCP1 and AtUCP2 Transport Aspartate and Glutamate92.3. The Human Glutamate Carriers102.4. Arabidopsis BOU and Yeast Ymc2p Transport Glutamate112.5. Human UCP2 Transports Aspartate123. Mitochondrial Carriers for Basic Amino Acids133.1. The Human and Yeast Ornithine Carriers133.2. Human SLC25A29 Transports Basic Amino Acids with a Preference for Lysine and Arginine 153.3. Arabidopsis BAC1 and BAC2154. Mitochondrial Carriers for Neutral Amino Acids164.1. The Human and Yeast Glycine Carriers165. Future Perspectives17

## 1. Introduction

Mitochondrial carriers (MCs) are eukaryotic nuclear-encoded membrane proteins that transport di- and tri-carboxylates, ketoacids, amino acids, nucleotides, coenzymes/cofactors, sulfate and phosphate across the mitochondrial inner membrane [1,2]. They constitute a family of proteins that contain distinctive and conserved sequence features arranged in three homologous tandem sequence repeats of about 100 amino acids [3]. Each repeat contains two hydrophobic transmembrane segments connected by a signature sequence motif PX[D/E]XX[K/R]X[K/R] (20–30 residues) [D/E]GXXXX[W/Y/F][K/R]G (PFAM PF00153) [4]. These sequence features have been used to recognize genes encoding MCs (in humans, called SLC25) in the genomes of various organisms. As a result, 53 members in humans, 35 in *Saccharomyces cerevisiae* and 58 in *Arabidopsis thaliana* [5,6] have been detected. Most MC genes encode proteins consisting almost exclusively of the three sequence repeats, but some encode longer MCs with an additional extensive domain at their N-terminus. MC family members can be divided into more than 25 subfamilies based on their substrate specificity and distinctive triplets [6,7].

Until now, the substrates transported by individual MCs have been identified using a strategy consisting of heterologous MC gene expression, recombinant protein purification and reconstitution into liposomes, followed by direct transport assays, known as the “expression-purification-reconstitution-assay” (EPRA) method [8]. Typically, this method begins with the recognition of an MC gene in sequence databases and cloning it into suitable expression vectors. Afterward, the MC protein is produced in a selected host, following expression trials in various strains of *Escherichia coli*, *S. cerevisiae* or other expression systems. Subsequently, the protein is purified and incorporated into phospholipid vesicles (i.e., liposomes) that are used in measurements of MC-mediated uptake or export of radioactively labelled substances [9]. Lastly, characterization of transport properties of the isolated MC in vitro is achieved by transport assays to determine substrate specificity, substrate affinity, kinetic parameters of transport, mode of transport (e.g., uniport, antiport or symport), and possible dependence on the proton gradient across the membrane. In kinetics experiments the transport reaction is stopped by using specific or unspecific inhibitors that block transport rapidly and completely [10,11]. Virtually all MCs have been found to catalyze an antiport (exchange) faster than the unidirectional (uniport) transport of substrates; in fact, most MCs are obligatory exchangers [12]. The results of transport assays define the activity of MCs and usually provide clues to their physiological roles in cells. 

**Figure 1:**
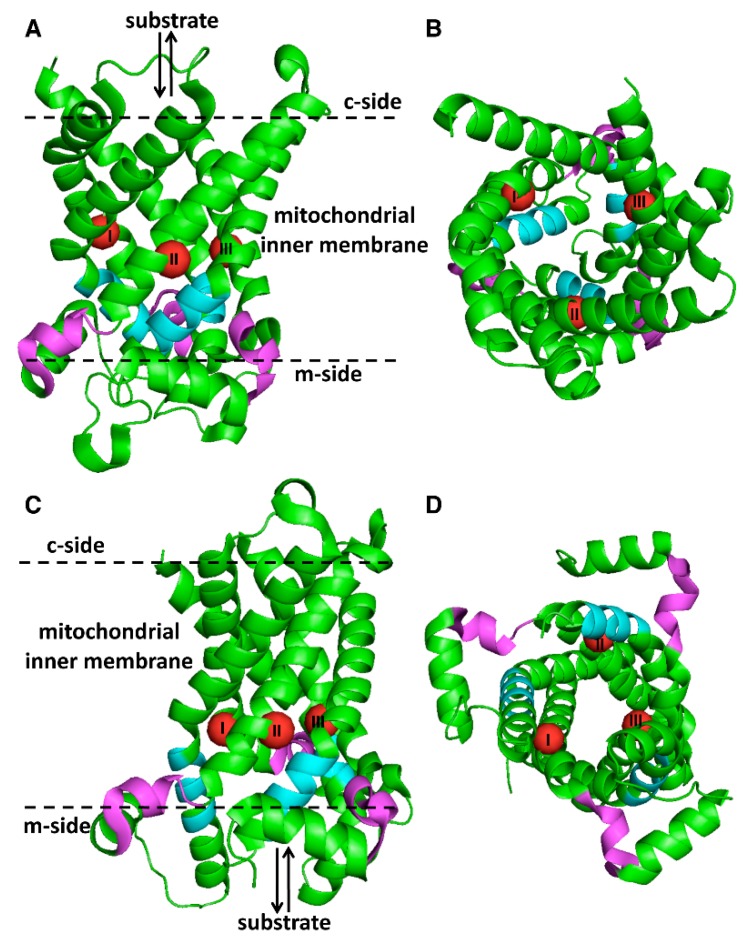
Structures of AAC. The carboxytractyloside-inhibited bovine AAC (PDB ID 1OKC) (**A**,**B**) and a bongkrekic acid-inhibited AAC of *Thermothelomyces thermophila* (PDB ID 6GCI) (**C**,**D**) are viewed laterally in the plane of the inner mitochondrial membrane (**A**,**C**), and from the c- (**B**) and m-sides (**D**). The first and second parts of the signature motif sequence are colored in cyan and magenta, respectively. Contact points I–III are shown as red spheres.

X-ray crystal structures of different versions and conformations of the ADP/ATP carrier (AAC) have contributed to understanding how this, and most likely the other members of the MC family function [13,14,15]. The structures demonstrate that the three tandem sequence repeats form a single three-fold symmetric domain, designated the “MC domain.” This domain consists of a bundle of six transmembrane α-helices around a substrate translocation pore and three short α-helices almost parallel to the plane of the membrane on the matrix side in the loops connecting the transmembrane α-helices 1 and 2, 3 and 4, and 5 and 6 (Figure 1). The substrate translocation pathway or pore is closed alternatively on the cytoplasmic (c) or matrix (m) side by a c-gate or m-gate, respectively (Figure 1). In the structures of AAC inhibited by carboxyatractyloside [13,14] the m-gate is closed (Figure 1A,B), partly by a salt bridge network formed by the charged residues of the first part of the signature motif sequence (PX[D/E]XX[K/R]X[K/R] of the odd-numbered transmembrane helices, whereas the c-gate is open. On the contrary, in the structure of the bongkrekic acid-inhibited AAC [15] the m-gate is open and the c-gate is closed (Figure 1C,D), partly by the contribution of another salt bridge network ([D/E]XX[K/R], which is less conserved and found towards the end of the even-numbered transmembrane helices). Carboxyatractyloside and bongkrekic acid had been shown to access AAC from the c- and m-sides, respectively, and bind (as the carrier substrates ADP and ATP do) to a single binding site at the center of the carrier, locking it in conformations similar to (but not exactly the same) the c- and m-states, respectively [16,17]. Consequently, the single binding site is exposed to the c- and m-sides alternatively. Furthermore, upon substrate binding, AAC has been shown to undergo a conformational change from the c- to the m-state, or *vice versa*, via an intermediate or transition state (i.e., the conformation of the carrier with the substrate bound) [16,17,18]. Based on sequence and structure analyses, and the substrate specificity of characterized MCs until 2006, the residues of three specific sites (contact points) located between the two gates at the same level in the membrane plane and protruding into the pore have been proposed to participate in substrate binding (Figure 1) [19]. Contact point II residues are thought to discriminate between the main substrate classes of MCs; i.e., for nucleotides (G[IVLM]), carboxylic acids (R[QHNT]) and amino acids (R[DE]). In the case of the latter group the positively and negatively charged residues of contact point II would interact with the C_α_ carboxylate and amine of all the amino acid substrates, whereas subfamily-conserved residues of contact point I would coordinate the other end of the substrates (in these carriers contact point III usually consists of an almost invariable arginine). Therefore, the substrate binding site is similarly located in all MCs at the center of the substrate translocation pore. However, considering the numerous members of the MC family and the highly variable size and chemical characteristics of their specific substrates, other residues located in proximity to the contact points necessarily contribute to form the binding site [6,20]. MC structures support and provide the molecular details of the previously proposed “single binding center-gating pore” transport mechanism for substrate exchange based on antiport transport experiments with AAC, and an understanding of the inhibitory effects of carboxyatractyloside and bongkrekic acid [16]. In this “ping-pong” transport mechanism [14,16,17,21], the substrate enters the central binding site, for example from the c-side of the membrane, into an MC with the c-gate open and the m-gate closed (Figure 1A,B). Substrate binding induces conformational changes that trigger closing of the c-gate and opening of the m-gate (Figure 1C,D), hence the substrate leaves its binding site on the m-side, rendering the empty binding site accessible to another substrate from the m-side to achieve a translocation reaction in the opposite direction and complete the substrate exchange cycle. In line with this mechanism, the major conformational differences between the carboxyatractylside- and bongkrekic acid-inhibited structures are found around the contact points, where substrate binding is supposed to trigger the transformation of the c-state into the m-state and *vice versa*.

To understand the functional role of MCs in vivo, other information is used, such as subcellular localization, regulation (direct or at transcriptional level), expression levels in various tissues, complementation of phenotypes and the effects of their knockdown, knockout, overexpression and disease-causing mutations, along with the conclusions drawn from transport assays concerning, for example, substrate specificity and transport mode. These clues can be combined with the knowledge of metabolic pathways (related to the identified substrates) and their compartmentalization in cells of different tissues to suggest a plausible physiological function of mitochondrial carriers. 

**Figure 2:**
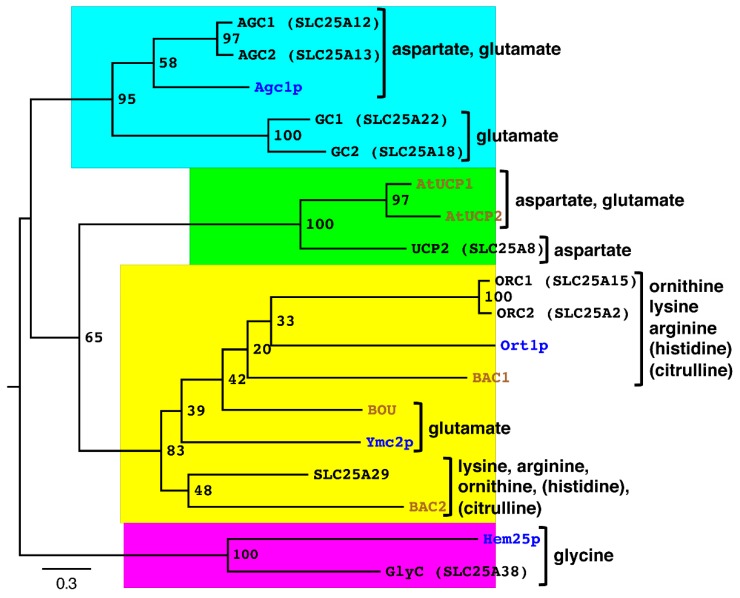
Phylogenetic tree of biochemically characterized mitochondrial carries that transport amino acids. The tree was constructed using PhyML v3.1 from a multiple-sequence alignment with ClustalW in Seaview4 and drawn in FigTree v1.4.2. Bootstrap values for 1000 replicates are reported on each node. The names of mitochondrial carriers (MCs) from humans, *Arabidopsis thaliana* and *Saccharomyces cerevisiae* are shown in black, brown and blue, respectively. The clusters are color coded as in Table 1. The transported amino acid substrates for each carrier group are indicated with those that vary between one carrier and another in parentheses: histidine is not transported by ORC1 or Ort1p; citrulline is not transported by Ort1p, BAC1 or SLC25A29. AtUCP1 and AtUCP2 also transport malate, oxaloacetate, 2-oxoglutarate, oxalate, succinate, phosphate, sulfate and thiosulfate. Human UCP2 also transports malate, oxaloacetate, phosphate and sulfate.

Mitochondria have a central role in amino acid metabolism based on the following facts: (i) many of the carbon skeletons used in amino acid biosynthesis originate from metabolites of the tricarboxylic acid (TCA) cycle, and (ii) the carbon backbone of all amino acids may be converted, directly or indirectly, into TCA cycle intermediates. In addition, key reactions for the removal of ammonium ions generated from the catabolism of amino acids take place in mitochondria. Consistently, MCs play important roles in the import of amino acids and the export of precursors for biosynthetic pathways, not only of amino acids but also of other important metabolites. Furthermore, the imported amino acids may be used in mitochondrial translation. Until now, 18 MCs that transport amino acids in humans, *S. cerevisiae* and *A. thaliana* have been identified (Figure 2), characterized and shown to be localized to the mitochondrial inner membrane. They can be divided into MCs that transport acidic, basic and neutral amino acids. In this review the transport properties and physiological functions of these MCs and the available structural information about them will be discussed.

## 2. Mitochondrial Carriers for Acidic Amino Acids

### 2.1. The Human and Yeast Aspartate-glutamate Carriers

Two MCs for aspartate and glutamate, referred to as AGC1 (SLC25A12 or Aralar1) and AGC2 (SLC25A13 or citrin), have been identified in humans [22]. These two proteins are different from the great majority of MCs, as they possess an N-terminal domain of about 300 residues in front of the C-terminal MC domain. The N-terminal domains of AGC1 and AGC2 protrude into the intermembrane space of mitochondria, contain several EF-hand Ca^2+^-binding motifs and are the regulatory portions of the proteins. AGC1 and AGC2 are isoforms with 78% sequence identity; with the exclusion of their N-terminal domains, sequence identity is 88% (Table 1). Full-length AGC1 and AGC2 and the AGC1- and AGC2-MC domains, were expressed in *E. coli* and tested with the EPRA method; all four proteins were found to transport the acidic amino acids aspartate, glutamate and cysteinsulfinate with high specificity [22]. In the characterization of AGC1 and AGC2 by the EPRA method, pyridoxal 5’-phosphate and bathophenanthroline were used as inhibitors of the carrier activity. Unfortunately, for these carriers, and for all the others described in this review, no specific and powerful inhibitor, such as atractyloside and bongkrekic acid for AAC, is known of yet. AGC1 and AGC2 are strict antiporters; and while aspartate and cysteinsulfinate are transported with a net negative charge, glutamate is co-transported with a proton, making the aspartate/glutamate or cysteinsulfinate/glutamate exchanges electrophoretic [22]. For this reason, in energized mitochondria, AGC1 and AGC2 catalyze the efflux of aspartate and the influx of glutamate, and the malate-aspartate shuttle is unidirectional. The K_m_ values of both AGC1 and AGC2 are approximately 0.05 and 0.2 mM for aspartate and glutamate uptake, respectively, and the V_max_ is about four times higher for AGC2 (~200 mmol/min/g protein) than for AGC1 [22]. Contact point II of AGC1 and AGC2 contains an RD motif, which is typical of MCs for amino acids; contact point I contains two lysines, which are likely candidates to bind the negative charge of the substrate side chain (Figure 1). The activities of AGC1 and AGC2 are regulated by the concentration of Ca^2+^ in the cytosol, which binds the EF-hand motifs in the N-terminal regulatory domain [22,23]. Recently, the crystal structures of the soluble N-terminal domains of AGC1 and AGC2 in the presence and absence of Ca^2+^ have been solved [24]. Surprisingly, these structures revealed that EF-hands 4–8 (of eight in total) form static homodimers, and EF-hands 1–3 undergo Ca^2+^-induced conformational changes, and bind an amphipathic α-helix in the short C-terminal extension of the MC domain. It has been hypothesized that in the absence of Ca^2+^ the accessibility of the substrate binding site (in the MC domain) from the cytoplasmic side is blocked by the N-terminal domains of AGC1 and AGC2, whereas the Ca^2+^-induced conformation of the N-terminal domains and their consequent interaction with the C-terminal amphipathic α-helix allow the substrates to access the binding site and be transported. 

**Figure 3:**
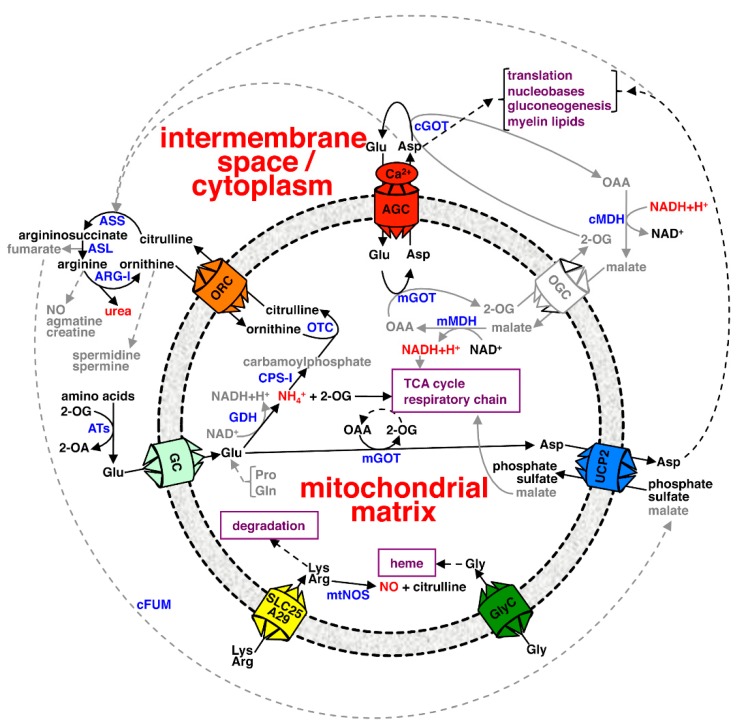
The metabolic roles of MCs transporting amino acids. The schemes do not show all the metabolic pathways in which the individual carriers are involved. The carriers for amino acids are in color, whereas the oxoglutarate carrier is not colored. Substrates are abbreviated in black/gray: 2-OA, 2-oxoacid; 2-OG, 2-oxoglutarate; OAA, oxaloacetate. Enzymes are abbreviated in blue: ARG-I, arginase I; ASL, argininosuccinase; ASS, argininosuccinate synthase; ATs, amino transferases; cGOT and mGOT, cytoplasmic and mitochondrial glutamate oxaloacetate transferase; cFUM, cytoplasmic fumarase; cMDH and mMDH, cytoplasmic and mitochondrial malate dehydrogenase; CPS-I, carbamoyl phosphate synthetase I; GDH, glutamate dehydrogenase; mtNOS, mitochondrial nitric oxide synthetase; OTC, ornithine transcarbamoylase.

While AGC2 is ubiquitous and abundantly expressed in liver, AGC1 is present at high levels in heart and skeletal muscle, and the brain [25,26,27,28]. Wherever expressed the AGCs are involved in the malate-aspartate shuttle, which transfers the reducing equivalent of NADH from the cytosol to the mitochondrial matrix. The malate-aspartate shuttle is one of the systems that compensate for the lack of mitochondrial transporters for direct transfer of NADH, formed in cytoplasmic catabolic pathways, to the matrix, where it is oxidized by complex I of the respiratory chain. In this shuttle, the net transport of the reducing equivalent of NADH into the matrix is accomplished by the OGC-catalyzed 2-oxoglutarate/malate exchange, the AGC-catalyzed aspartate/glutamate exchange and two enzymatic reactions in the cytoplasm and matrix (Figure 3) [29,30]. Moreover, AGCs play an important role in the mitochondrial export of aspartate, which in the cytosol is used in translation, and as a precursor for the biosynthesis of metabolites: nucleobases (probably involving both AGCs in most cells), N-acetyl-aspartate used in the formation of myelin lipids (mainly AGC1 in brain), glucose via gluconeogenesis (AGC2 in liver) and urea formed in the urea cycle (AGC2 in liver) (Figure 3). Given that AGCs transport cysteinsulfinate, these transporters are also involved in sulfur metabolism, as cysteinsulfinate is an intermediate in cysteine catabolism. Due to their central roles in the above-mentioned processes and the presence of N-terminal EF-hand motifs, AGCs are important targets in the coordinated regulation of ureogenesis, gluconeogenesis and amino acid catabolism by Ca^2+^ [23].

Support for the important physiological roles of AGC1 and AGC2 is provided by the features of the genetic diseases associated with these transporters and by expression depletion experiments in cell cultures. Two mutations in the human AGC1 gene that lead to marked reduction in transport function and AGC1 deficiency (OMIM 612949) have been reported [31,32]. The disease symptoms are infantile epilepsy, congenital hypotonia and developmental delay, in conjunction with hypomyelination and reduced N-acetyl-aspartate in the brain. A lack of functional AGC1, which is the predominant isoform in neurons, diminishes cytosolic aspartate leading to reduced biosynthesis of N-acetyl-aspartate, purines and pyrimidines, along with reduced translation; all may contribute to the observed symptoms [33]. The AGC1 deficiency phenotype was also documented by studies in AGC1 knockout mice, which displayed growth retardation; impaired central nervous system functioning, due to reduced brain levels of N-acetyl-aspartate and myelination [34]; and impaired proliferation of brain precursor cells [35].

Mutations in the gene encoding AGC2 are known to cause two liver diseases—neonatal intrahepatic cholestasis by citrin deficiency (NICCD, OMIM 605814) and type-II citrullinemia (CTLN2, OMIM 215700)—mainly due to lack of cytosolic NADH oxidation and impaired urea cycle functioning [36]. The main symptoms of NICCD are transient intrahepatic cholestasis, hepatomegaly, growth retardation, citrullinemia, ketotic hypoglycemia, hypoproteinemia and elevated levels of methionine, threonine, tyrosine, phenylalanine, lysine and arginine [37,38]. After one or a few years of age, the patients with this phenotype usually seem healthy but may go on to develop CTLN2 in adulthood. CTLN2 is characterized by citrullinemia, hypoproteinemia and hyperammonemia, which leads to encephalopathy and neuropsychiatric symptoms [26,39,40]. Furthermore, AGC2 expression is co-regulated with components of the urea cycle, which again supports the role of AGC2 in providing cytosolic aspartate for urea synthesis [41]. It has also been shown that the expression of AGC2 is upregulated by the transcription factors: USF1, which is consistent with the role of this protein, which is providing NAD for glycolysis, and FOXA2, which may be partly responsible for the high expression of AGC2 in liver [42]. The expression of AGC1 in neuronal cells is regulated by CREB [43], which may also be involved in the epigenetic upregulation of AGC1 expression in hepatocellular carcinoma [44]. Additional support for the role of AGC1 in the malate-aspartate shuttle stems from the observation that glucose-induced oxidative metabolism is reduced upon the depletion of AGC1 expression in insulin-secreting cells [45]. 

Interestingly, a yeast ortholog of the human AGCs, Agc1p (YPR021cp), has also been characterized [46], whereas no close homologs have been found in plants (Figure 2). Agc1p displays identical contact point residues as its human counterparts; similar substrate specificity; and a long N-terminal extension before the C-terminal MC domain, which, however, does not contain Ca^2+^-binding motifs. The MC domain of Agc1p shares 57%–58% sequence identity with the MC domains of human AGCs (Table 1). Varying from human AGCs, Agc1p is not a strict antiporter; i.e., it may also catalyze the unidirectional transport of glutamate or aspartate [46]. In the characterization of Agc1p by the EPRA method, pyridoxal 5’-phosphate and HgCl_2_ were used as inhibitors of the carrier activity. It has been suggested that Agc1p is involved in the mitochondrial import of glutamate to be used as a source of carbon and nitrogen for ornithine synthesis, and in the malate-aspartate NADH shuttle, which is critical for growth on acetate and fatty acids [46]. Furthermore, it has been proposed that Agc1p (or Ymc2p) catalyzes the export of mitochondrially synthesized glutamate in yeast grown in synthetic minimal medium (without glutamate) supplemented with non-fermentable carbon sources [47,48].

### 2.2. Arabidopsis AtUCP1 and AtUCP2 Transport Aspartate and Glutamate

Although no ortholog of the human AGCs has been found in Arabidopsis based on sequence similarity, two MCs previously named uncoupling proteins, AtUCP1 (PUMP1 or AT3G54110) and AtUCP2 (PUMP2 or AT5G58970), have recently been shown to transport aspartate, glutamate and cysteinsulfinate [49]. These two carriers share 74% sequence identity in their MC domains, and differing from AGCs, they lack the N-terminal domain with Ca^2+^-binding motifs and the negatively charged residue typical of MCs for amino acids in contact point II (which consists of an RN motif). An arginine in contact point I could potentially be involved in the binding of the terminal negative charge of the amino acid substrate side chain (Figure 1). AtUCP1 and AtUCP2 are instead more closely related to human UCP2 (Figure 2 and Table 1); for UCP2 see Section 2.5. Besides aspartate, glutamate and cysteinsulfinate, and AtUCP1 and AtUCP2 transport the acidic amino acids cysteate and α-aminoadipate, and to a much lesser extent, the neutral amino acid cysteine. In addition, these two MCs are capable of transporting other types of substrates to various degrees: dicarboxylates (e.g., malate, oxaloacetate, 2-oxoglutarate, oxalate and succinate) and inorganic anions (e.g., phosphate, sulfate and thiosulfate). The K_m_ values of the two proteins for aspartate and glutamate are rather high (about 0.8 and 2 mM, respectively), while the V_max_ of AtUCP1 (about 30 mmol/min/g protein) is about six to seven times higher than that of AtUCP2. The K_m_ values of AtUCP1 and AtUCP2, which are considerably higher than those of the AGCs, might be the cost for having a broader substrate specificity. Regarding the inhibitors, the transport activities of AtUCP1 and AtUCP2 are inhibited more than 90% by pyridoxal 5’-phosphate, bathophenanthroline and tannic acid when used separately; mercurials inhibited AtUCP1 more than AtUCP2, whereas the dicarboxylate analogues butylmalonate and phenylsuccinate inhibited both to various degrees. 

AtUCP1 is located in mitochondria and has been implicated in photorespiration [50]. This process requires mitochondria and peroxisomes to accomplish the glycolate pathway, and in particular, the mitochondrial enzyme glycine decarboxylase, which reduces NAD^+^ to NADH, and peroxisomal hydroxypyruvate reductase, which oxidizes NADH to NAD^+^. Therefore, it was proposed that AtUCP1, and possibly AtUCP2, are involved in the mitochondrial export of the reducing equivalents of NADH, which could enter into peroxisomes for oxidation and be recycled back into mitochondria (see [49], Figure 10). This hypothesis implies that AtUCP1 contributes to the mitochondrial malate-aspartate shuttle with an opposite transfer of the NADH-reducing equivalents compared to that which occurs in mammals. Although AtUCP1 and AtUCP2 are capable of catalyzing both the antiport transport reactions required for an inverted malate-aspartate shuttle, i.e., the aspartate/glutamate and malate/2-oxoglutarate exchanges, other MCs are capable of transporting 2-oxoglutarate and malate in plants. Therefore, AtUCP1 and AtUCP2 most likely export glutamate from the mitochondrial matrix in exchange for cytoplasmic aspartate; the cellular concentrations of both amino acids are higher than those of the dicarboxylates that are transported by these proteins. Worth noting is that the aspartate/glutamate exchange catalyzed by human AGC1 and AGC2 is electrophoretic [22], whereas the aspartate/glutamate exchange catalyzed by AtUCP1 and AtUCP2 is electroneutral [49], and therefore, not dependent on the proton motive force (ΔμH^+^). 

AtUCP1 and AtUCP2 can also be involved in nitrogen metabolism owing to their ability to exchange amino acids for various dicarboxylates. Given that nitrogen often limits plant growth and that ammonium (released from various reactions in different cellular compartments) is recycled in plastids, AtUCP1 and AtUCP2 could be involved in shuttling ammonium ions outside mitochondria by exporting asparate or glutamate in exchange for their nitrogen-free substrates. For example, they might export glutamate, formed from ammonium and 2-oxoglutarate, in exchange for 2-oxoglutarate [49]. 

### 2.3. The Human Glutamate Carriers 

The closest relatives to AGC1 and AGC2 in humans are the two isoforms of the glutamate carrier, known as GC1 (SLC25A22) and GC2 (SLC25A18) (Figure 2), which, however, lack the N-terminal regulatory domains of AGCs. GC1 and GC2 share 68% identical sequence and display 46% sequence identity with the MC domains of AGC1 and AGC2 (Table 1). The charged residues of the contact points of GC1 and GC2 are identical to those of AGCs, with the exception that GCs lack arginine in contact point III (Figure 1). The GCs are conserved in most multicellular animals but appear to be lacking in yeast or plant genomes. 

GC1 is mainly expressed in the pancreas, liver and brain, and to a lesser extent in other tissues. GC2 is expressed in most tissues and always at a lower level than GC1, with the exception of the brain, where both isoforms are expressed at an equal level [51]. An EPRA-method study found that GC1 and GC2 almost exclusively transport glutamate; their K_m_ values for glutamate uniport uptake are approximately 5 and 0.3 mM, respectively, and their V_max_ values about 12 and 4 μmol/min/g protein, respectively [51]. In glutamate/glutamate antiport experiments, the K_m_ values were virtually identical, but the V_max_ values increased about 10-fold. When a pH gradient was applied across the proteoliposomal membrane (acidic outside), the rates of GC1- and GC2-catalyzed glutamate uptake (but not the glutamate/glutamate exchanges) increased four- to five-fold, whereas the K_m_ values were unaffected. These observations clearly indicate that the translocation of glutamate across the proteoliposomal membrane requires the co-transport of protons [51]. Pyridoxal 5’-phosphate and bathophenanthroline were used as inhibitors of GC1 and GC2 in the EPRA method but also tannic acid and bromocresol purple efficiently inhibit their transport activity. Two GCs from *Drosophila melanogaster* have also been characterized and shown to have transport properties similar to those of their human counterparts [52]. In comparison to AGCs that catalyze the Ca^2+^-regulated antiport exchange of glutamate^−1^ and H^+^ for aspartate^−1^, GCs catalyze a symport of glutamate^−1^ and H^+^. Based on these considerations, it was proposed that GCs catalyze a ΔμH^+^-dependent import of glutamate into the mitochondrial matrix [51]. Here, glutamate is transformed into ammonia and 2-oxoglutarate by glutamate dehydrogenase, which in mammals is localized exclusively in mitochondria (Figure 3). In liver and kidney cells, where GC1 is most predominant, ammonia (accumulated in the organism in the form of glutamine and alanine) is detoxified by the urea cycle (which initiates in the mitochondrial matrix), while the majority of formed 2-oxoglutarate is used in gluconeogenesis [53]. In non-hepatic tissues, where GC1 and GC2 may contribute to mitochondrial glutamate import to different degrees, 2-oxoglutarate is mainly fed into the TCA cycle for further oxidation and the production of ATP by oxidative phosphorylation. However, in pancreatic β-cells incubated with an excess of glucose, GC1 was shown to catalyze the mitochondrial export of glutamate, which in the cytoplasm, is important for glucose-stimulated insulin secretion [54].

GC1 deficiency causes early infantile epileptic encephalopahty type 3 (EIEE3, OMIM 609304), which is manifested by erratic refractory seizures, usually myoclonic, with an electroencephalogram (EEG) showing a suppression-burst pattern [55,56,57,58]. Other symptoms include hypotonia, microcephaly, abnormal visually evoked potentials, migrating partial seizures, spasticity and cerebral atrophy. The etiopathology of the disease is not well understood. However, it has been suggested that EIEE3 patients have altered cellular concentrations of glutamate, which in the brain is an important neurotransmitter; therefore, neurological symptoms are mainly associated with this disease [55,59].

### 2.4. Arabidopsis BOU and Yeast Ymc2p Transport Glutamate

No apparent GC ortholog is found in plants or yeast. However, recently the MCs BOU (“a bout de souffle” or AT5G46800) and Ymc2p (YBR104wp) from *A. thaliana* and *S. cerevisiae*, respectively, have been shown to transport glutamate [60]. The MC domains of BOU and Ymc2p share 41% identical protein sequence, and very peculiarly, have much higher sequence identity with characterized MCs for basic amino acids than for those transporting glutamate (Table 1). The sequence similarities between BOU and Ymc2p and all the other MCs explain why these two carriers cluster together with the basic amino acid carriers, and why the bootstrap values in this cluster are lower (the yellow group in Figure 2). These observations suggest that BOU and Ymc2p may originate from an MC ancestor in common with the basic amino acid carriers, whereas GCs derive from an MC ancestor in common with AGCs (Figure 2). The separate evolutionary trails of these two groups of MCs for glutamate are consistent with the different metabolic context of GCs in animals (Section 2.3) and BOU and Ymc2p in plants and yeast, as discussed below. Another peculiar sequence feature is that Ymc2p lacks the negatively charged residue in contact point II, whereas BOU has an RE motif, which is typical for MCs transporting amino acids (Figure 1). In addition, unlike all the other characterized MCs transporting aspartate and/or glutamate, both BOU and Ymc2p lack a positively charged residue in contact point I (thought to interact with the negatively charged substrate side chain). Therefore, it is not yet completely clear how BOU and, even more so, Ymc2p bind glutamate.

Characterization of BOU and Ymc2p with the EPRA method showed that, besides glutamate, these MCs transport homocysteinesulfinate, although to a lesser extent [60]. The K_m_ values of BOU and Ymc2p for glutamate were determined to be about 25 and 15 μM, respectively, while the V_max_ of both carriers was approximately 4 μmol/min/g protein for glutamate uniport and three to five times higher for the glutamate/glutamate antiport. It is noteworthy that the V_max_ values for glutamate of BOU and Ymc2p are in the same range as those of GCs (and many times lower than those of AGCs and AtUCP1-2), whereas the K_m_ values are less than one tenth of those of GCs. Similar to GCs, BOU and Ymc2p catalyze a glutamate and proton symport, suggesting that the uptake of glutamate is favored in energized mitochondria. The growth defects of *S. cerevisiae* devoid of the genes for Ymc2p and Agc1p (the two MCs known to transport glutamate in yeast) are complemented by the recombinant expression of Agc1p, Ymc2p, or BOU [60]. These results are in agreement with the conclusion that all three carriers are capable of similar transport functions. Moreover, it was proposed that the poorly expressed Ymc2p-paralog, Ymc1p, which shares 65% sequence identity with Ymc2p, also transports glutamate [60]. Consistently with this proposal, Ymc1p and Ymc2p have previously been suggested to play a role in glutamate metabolism in yeast [61].

Unlike in animals, where digested proteins are one of the main energy sources for ATP production, in yeast and plants, amino acids are rarely oxidized for energy conversion; moreover, ammonium is generally recycled due to a shortage of nitrogen under normal conditions. Besides the basic protein/amino acid turnover that takes place in all types of cells and organisms, in plants and yeast amino acid oxidation occurs only under certain growth conditions and in specific metabolic states [62]. In these circumstances, BOU and Ymc2p are likely to contribute to the mitochondrial import of glutamate, which may be oxidized by various isoforms of glutamate dehydrogenase that exist in the mitochondria of *A. thaliana* and *S. cerevisiae* [60]. Furthermore, matrix glutamate supplied by BOU and Ymc2p may also be used for tetrahydrofolate (THF) biosynthesis (in Arabidopsis) and THF polyglutamylation (in both yeast and Arabidopsis). THF is an important cofactor in one-carbon metabolism; it is required in yeast for serine hydroxymethyltransferase (SHMT) and in Arabidopsis for glycine decarboxylase and SHMT. In line with this role of BOU in Arabidopsis, BOU knockout causes glycine accumulation, due to reduced glycine decarboxylase activity and consequent photorespiration defects [63]. Under specific conditions BOU and Ymc2p may also operate in the reverse direction, exporting glutamate, which is an important step in ammonium fixation in yeast [48]. 

### 2.5. Human UCP2 Transports Aspartate 

Human UCP2, as well as other MCs, was originally named uncoupling protein due to its sequence homology with UCP1. UCP1 was demonstrated to be essential for thermogenesis as an H^+^ carrier, given its ability to uncouple the proton gradient across the mitochondrial inner membrane from ATP synthesis in brown adipose tissue [64,65]. However, with the exception of mammalian UCP1, the other members of the “UCP subfamily” of MCs have at most displayed mild uncoupling effects. In phylogenetic trees, the “UCP subfamily” clusters together with or close to the dicarboxylate carrier subfamily; in fact, some of the Arabidopsis UCPs (AtUCP4-6) have been shown to be dicarboxylate carriers [6,66]. Human UCP2 was the first identified carrier in the UCP cluster capable of transporting an amino acid, i.e., aspartate [67], followed by AtUCP1 and AtUCP2, that were shown to transport mainly aspartate and glutamate (see Section 2.2) [49]. These three carriers cluster separately from the other MCs for amino acids (Figure 2) and are likely to have a more recent common ancestral protein with the dicarboxylate carriers than with the AGCs and GCs [6]. Furthermore, all three proteins have RN in contact point II, which is more similar to R[QHNT] found in MCs for carboxylates than to R[DE] found in most MCs for amino acids (Figure 1). In addition, UCP2 has positively charged residues in proximity to contact point I that could bind the β-carboxyl group of aspartate. Very recently, human UCP5 and UCP6, that have RA in contact point II, have also been shown to transport aspartate (and UCP5 glutamate as well), although these substrates are translocated to a much lesser extent than sulfur oxyanions, phosphate and dicarboxylates [68].

Investigation of the transport properties of UCP2 with the EPRA approach, utilizing the inhibitors pyridoxal 5’-phosphate and bathophenanthroline, has shown that this protein transports dicarboxylates/inorganic anions, such as malate, oxaloacetate, phosphate and sulfate, in addition to aspartate [67]. Results further suggest that the UCP2-mediated transport of phosphate is proton-coupled, which would favor mitochondrial import of phosphate in exchange for aspartate or a dicarboxylate in energized mitochondria. The proposed physiological role of UCP2, which is expressed in many human tissues, is to export four-carbon-metabolites from the mitochondrial matrix in exchange for cytosolic phosphate. This is accomplished in (i) cell metabolic reprogramming from oxidative phosphorylation towards aerobic glycolysis and in (ii) glutamine oxidation for energy metabolism [67,69]. In the first case, increased expression of UCP2 would lead to increased mitochondrial export of malate or oxaloacetate and, consequently, to reduced activity of the TCA cycle, respiratory chain and oxidative phosphorylation, which in turn would reduce the ATP/ADP ratio and reactive oxygen species. In the second case, UCP2 would export aspartate formed by the action of glutamate oxaloacetate aminotransferase, while 2-oxoglutarate (the other product of the reaction) would be transformed into oxaloacetate by the enzymes of the TCA cycle (Figure 3). That way, electrons are fed into the respiratory chain for ATP production, and cytosolic aspartate may be used as a precursor for gluconeogenesis, and for protein and nucleotide biosynthesis. The hypothesis that UCP2 is at the crossroads of a metabolic switch is supported by the fact that mutations in UCP2 cause hyperinsulinism [70,71,72] and that increased UCP2 expression is associated with a reduced risk of obesity and type-2 diabetes in humans [73]. UCP2 could also be involved in the aspartate-argininosuccinate shunt of the TCA/urea cycle by exchanging cytosolic malate, formed from fumarate by cytosolic fumarase [74], with mitochondrially produced aspartate to be used in the cytosolic portion of the urea cycle. 

## 3. Mitochondrial Carriers for Basic Amino Acids

### 3.1. The Human and Yeast Ornithine Carriers 

The human ornithine carriers ORC1 (SLC25A15) and ORC2 (SLC25A2) are two isoforms with 88% identical protein sequences in their MC domains (Table 1). Their closest relative in yeast is Ort1p (33% and 31% identical to ORC1 and ORC2, respectively) (Figure 2 and Table 1), which has the same charged residues in its contact points as ORC1, as discussed in detail below. Ort1p was the first MC to be identified as an ornithine transporter [75]; indeed, recombinant Ort1p was demonstrated to transport ornithine in exchange for protons, arginine or lysine. In the EPRA-method characterization, pyridoxal 5’-phosphate and bathophenanthroline were used as inhibitors, but N-ethylmaleimide, p-hydroxymercuribenzoate, p-chloromercuribenzene sulfonate and mersalyl are also effective inhibitors of Ort1p. Its primary physiological role was suggested to be the export of mitochondrial ornithine into the cytosol, where ornithine is used for arginine biosynthesis [75]. This suggestion concurred with the previous observation that a *S. cerevisiae* strain with a mutation in the gene for Ort1p is dependent on arginine supplementation for normal growth [76]. 

Human ORC1 and ORC2 mRNAs are found in the liver, pancreas, lung, testis, small intestine, spleen, kidney, brain and heart, with the former carrier always being expressed at higher levels [77]. Both isoforms transport the positively charged amino acids ornithine, lysine and arginine, and the neutral amino acid citrulline [77]. In addition, ORC2 transports the basic amino acids histidine, homoarginine, mono-methylarginine and asymmetric dimethylarginine (ADMA) (but not symmetric DMA), and the D-forms of lysine, arginine, ornithine and histidine [77,78]. The K_m_ values for ornithine are approximately 0.2 and 0.4 mM for ORC1 and ORC2, respectively, and the V_max_ values for the ornithine/ornithine homoexchange are 3 and 1.2 mmol/min/g protein, respectively. The V_max_ values of both proteins for lysine and arginine homoexchanges are within the same ranges as those for ornithine, whereas the K_m_ values of ORC1 for lysine and arginine are about 0.8 and 1.6 mM, respectively, and those of ORC2 0.3 and 0.7 mM, respectively. Pyridoxal 5’-phosphate was used as an inhibitor of ORC1 and ORC2 in the EPRA method, but their activities are also substantially inhibited by mercurials and N-ethylmaleimide. The residues determining the different substrate specificities of ORC1 and ORC2 have been investigated thoroughly by site-directed mutagenesis of the contact point residues and characterization of their effects on the transport properties [79]. While ORC1 has the typical RE motif in contact point II, ORC2 has QE (Figure 1). When the arginine in contact point II of ORC1 was swapped with glutamine (as in ORC2) the substrate specificity became similar to that of ORC2 and the V_max_ for ornithine homoexchange was decreased 13-fold. *Vice versa*, when glutamine in contact point II of ORC2 was changed to an arginine, the substrate specificity was converted into that of ORC1 and the V_max_ was increased 33-fold. These experiments suggest that the residues of contact point II of ORC1 and ORC2 bind the C_α_ carboxyl and amino groups of the substrate because the presence of the arginine in the first position of this contact point confers the protein with a stricter substrate specificity with more stereospecific selectivity and a higher turnover number compared to the protein having this position occupied by glutamine. Furthermore, other results provided evidence that glutamate in contact point I is the most likely candidate for interacting with the terminal amino group of the substrate side chain. In addition, a tryptophan on transmembrane α-helix four (W224 in both ORC1 and ORC2) was hypothesized to mediate interactions between contact points II, and III and thereby involve all three contact points in the conformational changes. 

ORC1 plays an important role in the urea cycle that mainly takes place in liver (Figure 3). Excess ammonia shuttled into mitochondria, usually in the form of glutamate by GCs (Section 2.3), is condensed with carbonate and phosphate from ATP to form carbamoylphosphate by carbamoylphosphate synthetase I. Subsequently, carbomoylphosphate is used in the conversion of ornithine into citrulline by ornithine transcarbamoylase, which is the only matrix-localized enzyme of the urea cycle. ORC1 is involved in the import of cytoplasmic ornithine in exchange for intramitochondrial citrulline [80,81,82]. In the cytosol, the remaining three enzymes of the urea cycle transform citrulline into ornithine and non-toxic urea, which is excreted. The expression level of hepatic ORC1 is under hormonal regulation and coordinated with the other enzymes of the urea cycle [83]. Moreover, in the small intestine, ORC1 and ORC2 are reckoned to be involved in the export of intramitochondrial citrulline (derived from glutamate, glutamine and proline) to be used for the biosynthesis of arginine in the kidneys and liver (Figure 3) [84,85]. 

Apart from being incorporated into proteins, arginine in specific cell-types is used as a precursor for the production of agmatine, creatine, spermine, spermidine and nitric oxide, and for reconversion into glutamate, glutamine and proline. The polyamines spermine and spermidine inhibit the two human ORC isoforms, which might suggest a feed-back inhibition regulatory mechanism [77]. Furthermore, the ORC2-mediated transport of ADMA across the mitochondrial membrane could serve two functions: (1) the export of ADMA generated from mitochondrial proteolysis into the cytoplasm for cytosolic degradation by dimethylarginine dimethylaminohydrolase (DDAH) or for export from the cell by plasma membrane transporters, and (2) the uptake of ADMA into mitochondria to enable cytosolic ADMA to be degraded by mitochondrial alanine-glyoxylate aminotransferase 2 (AGXT2) [78]. It is noteworthy that in respiring mitochondria, the unidirectional uptake of ADMA, which carries a positive charge, is favored by the electrical component (negative inside) of the proton motive force, compared to the unidirectional efflux from the mitochondria. However, ADMA can be exported (as well as imported) by ORC2 in exchange for the cationic amino acids lysine and arginine by an electroneutral exchange mechanism. ORC2 also transports mono-methylarginine [78], homoarginine [77] and perhaps di- and tri-methylarginines. Given that there are enzymes in the mitochondrial matrix that methylate various proteins [86] at the expense of S-adenosylmethionine imported into mitochondria [87,88,89], it is very likely that intramitochondrial mono-, di- and tri-methylarginines (derived from the proteolysis of proteins containing arginines with this specific post-translational modification) are exported from mitochondria to the cytoplasm. In contrast, the direction of homoarginine transport across the mitochondrial membrane in vivo is not well understood, because various indirect clues suggest that homoarginine biosynthesis and/or catabolism takes place in the mitochondrial matrix [85]. The fact that ORC2 catalyzes the transport of the D-forms of lysine, arginine, ornithine and histidine, besides their L-forms, demonstrating its inability to discriminate between these enantiomers [87], may be interpreted to indicate that ORC2 is involved in the metabolism of these cationic amino acid forms provided by various foods. 

The importance of ORC1 transport functions in human metabolism is underlined by HHH syndrome (hyperornithinemia-hyperammonemia-homocitrullinuria, OMIM 212138), which is caused by mutations in the SLC25A15 gene encoding ORC1 [36,77,90,91,92,93]. Defective ORC1 reduces the rate of the urea cycle, leading to hyperammonemia and to the build-up of ornithine in the cytosol, which causes hyperornithinemia, increased levels of polyamines and a secondary creatine deficiency (due to an inhibition of arginine-glycine amidotransferase (AGAT) by an excess of ornithine). Defective ORC1 also induces an increase in carbamoyl-phosphate, which in part condensates with lysine, forming homocitrulline by the action of ornithine transcarbamoylase, and in part, enters the pyrimidine pathway, explaining homocitrullinuria and increased excretion of orotic acid in urine. The clinical features of the syndrome, which usually start from the neonatal period to childhood and more rarely adulthood, are lethargy, episodes of confusion, coagulation abnormalities, hepatitis-like attacks, a progressive neurological dysfunction characterized by pyramidal signs (from hyperreflexia to spastic paraparesis), cerebellar signs (ataxia, poor motor coordination, tremor and seizures, mainly myoclonic), cognitive defects (e.g., intellectual disability), abnormal behavior and coma often upon consumption of a high-protein diet. Prescribed therapy consists of a low protein diet and supplementation with arginine and ammonia scavengers, which allow for an almost normal life expectancy. Interestingly, ORC2 may only partially compensate the lack of functional ORC1 in HHH syndrome patients, because it is much less expressed in liver and has a lower affinity for ornithine than ORC1.

### 3.2. Human SLC25A29 Transports Basic Amino Acids with a Preference for Lysine and Arginine

Another human MC that transports positively charged amino acids in the same cluster as the ORCs is SLC25A29 (Figure 2). The closest characterized relative of SLC25A29 is Arabidopsis BAC2 (basic amino acid carrier 2, with 40% identity, discussed in Section 3.3) (Table 1), whose substrates were identified prior to SLC25A29. Surprisingly, SLC25A29 is markedly distant from the other MCs with similar substrates, such as ORCs (31%–34%) and BAC1 (34% identity), when considering the percentage of identity with some MCs transporting negatively charged amino acids; e.g., AGCs (29%–33%), AtUCPs (30%–31%) and Ymc2p/BOU (38%–39%). It is worth noting that SLC25A29 has RE in contact point II but does not have a negatively charged residue in contact point I as most other MCs transporting basic amino acids have (Figure 1). 

SLC25A29 is expressed in heart, brain, liver, lung and kidney [94,95]. Biochemical characterization of SLC25A29 by the EPRA method demonstrates that it transports arginine and lysine, and to a much lesser extent, ornithine and histidine [96]. The transporter catalyzes high uniport rates—up to about one third of those of substrate exchange. The K_m_ of SLC25A29 was determined to be about 0.4 and 0.7 mM for arginine and lysine, respectively, and the V_max_ to be about 240 μmol/min/g protein for both substrates. In addition, SLC25A29 activity is inhibited by pyridoxal 5’-phosphate, tannic acid, HgCl_2_ and other mercurials.

Due to its ability to catalyze high rates of uniport transport, SLC25A29 is thought to be involved in the import of basic amino acids into the mitochondrial matrix for protein synthesis and amino acid degradation. The latter takes place mainly in liver and kidney under conditions of normal protein turnover, high protein content in the diet and starvation [96]. Intramitochondrial lysine is a substrate for the first enzyme of the saccharopine pathway (Figure 3), which is the major route for lysine degradation in upper eukaryotes [97]. Accordingly, in *Caenorhabditis elegans* it has been shown that a mutation of the gene corresponding to human SLC25A29 leads to a decreased generation of saccharopine [98]. Matrix arginine is also a substrate for mitochondrial nitric oxide synthase that converts arginine into citrulline and nitric oxide (Figure 3). This important signaling molecule can inhibit cytochrome c oxidase, be used for the production of peroxynitrite, or act as a signal in apoptosis and metabolic syndrome [99,100].

### 3.3. Arabidopsis BAC1 and BAC2

In Arabidopsis two MCs, BAC1 and BAC2, have been shown to transport basic amino acids [101,102,103]; both are found in the same cluster as the ORCs and BOU/Ymc2p (Figure 2). It is noteworthy that these two proteins share a markedly low sequence identity (29%) (Table 1). Surprisingly, BAC1 shares a higher identity (36%) with BOU. On the other hand, BAC2 is more similar to SLC25A29 with 40% identity, although it is also related to BOU (36% identity) and AGCs (30–33% identity). BAC1 and BAC2 also display differences in the contact point residues: the former has a glutamate and RE in contact points I and II, respectively, whereas the latter has no negatively charged residue and RD in the corresponding contact points (Figure 1). 

Purified recombinant BAC1 and BAC2 reconstituted in liposomes transport the positively charged amino acids arginine, lysine, histidine and ornithine, whereas only BAC2 transports neutral citrulline [101,103]. Pyridoxal 5’-phosphate and bathophenanthroline were used as inhibitors in the transport assays (but also HgCl_2_ and mersalyl block their activity). The K_m_ and V_max_ values of BAC1 for arginine are 0.19 mM and 48 μmol/min/g protein, respectively, and those of BAC2 are 0.16 mM and 38 μmol/min/g protein, respectively [103]. Thus, all the K_m_ values of the MCs for basic amino acids discussed herein are in the sub-millimolar range, whereas the V_max_ values vary from carrier to carrier.

Both BAC1 and BAC2 are expressed in the stem, leaf, flower, silique and seedling, with the highest levels of BAC1 in the seedling and BAC2 in the flower [101,102]. In seedlings, BAC1 and BAC2 have been suggested to catalyze the mitochondrial import of arginine, which is used as a nitrogen storage molecule before germination, and to produce ornithine and urea after germination through the action of mitochondrial arginase [101,103]. This hypothesis has been strengthened by the observation that overexpression of BAC2 causes arginine depletion and urea accumulation [104]. Moreover, matrix arginine may be used to stimulate seed germination, through nitric oxide produced by mitochondrial nitric oxide synthetase, and to synthesize glutamate, proline, polyamines, agmatine and alkaloids [103]. It has also been hypothesized that BAC2 in leaves catalyzes a citrulline/ornithine exchange to shuttle ammonia (which is produced in high amounts in mitochondria during photorespiration) to plastids where it is re-assimilated into glutamine [103]. In pollen, BAC2 has been suggested to export ornithine to the cytosol for the biosynthesis of proline, which is the most abundant amino acid in this tissue, and to play a role in drought stress protection [103]. These proposals are supported by the fact that BAC2 expression is stimulated by salt stress and leads to proline accumulation [105].

## 4. Mitochondrial Carriers for Neutral Amino Acids

### 4.1. The Human and Yeast Glycine Carriers

The MCs for glycine, human GlyC (SLC25A38) and yeast Hem25p (YDL119cp), are found in a phylogenetic cluster very distant from all the other MCs transporting the amino acids discussed above (Figure 2). Both GlyC and Hem25p, which share 35% identical sequence in their MC domains, have the second highest percentages of identity with Agc1p (27% and 29%, respectively). Although the evidence is weak, this might suggest that GlyC and Hem25p have a more recent evolutionary ancestor in common with Agc1p compared to other MCs, with 18%–26% and 21%–27% identity, respectively (Table 1). Furthermore, both GlyC and Hem25p have RD in contact point II and as one might guess from the absence of a charge in the substrate side chain, no charged residue in contact point I (Figure 1). 

Mutations in the human gene *SLC25A38* were found to give rise to congenital sideroblastic anemia, a disease characterized by mitochondrial iron overload in erythroblasts. This finding led to speculate that the MC SLC25A38 could be involved in heme and iron-sulfur cluster biogenesis, possibly by transporting glycine [106]. Later, a report based on findings obtained using the EPRA method provided evidence that GlyC and Hem25p transport glycine [107]. The yeast transporter was characterized in more detail; its K_m_ and V_max_ values for glycine/glycine antiport are 0.75 mM and 170 μmol/min/g protein, respectively. The initial transport rates for glycine antiport were about six times higher than those for glycine uniport. Hem25p transport activity was efficiently inhibited by pyridoxal 5’-phosphate, bathophenanthroline (both used in connection with the EPRA method), tannic acid and p-hydroxymercuribenzoate. Mitochondria isolated from a *Hem25* deletion strain displayed diminished glycine uptake, which was restored by reintroduction of the deleted gene. In addition, the *Hem25* deletion strain displayed a growth defect on non-fermentable carbon sources, decreased respiratory efficiency and diminished content of cytochrome bc1 complex, heme and cytochromes, which were all rescued by the recombinant expression of Hem25p or GlyC [107]. Based on these experiments, Hem25p and GlyC are considered to be responsible for the import of glycine into mitochondria (Figure 3), where glycine is transformed into the heme precursor δ-aminolevulinate. Subsequently, δ-aminolevulinate is exported to the cytoplasm by an unknown transporter to undergo further enzymatic transformations to form a porphyrin intermediate, which is shuttled back to the mitochondrial matrix to form the heme group. In yeast, glycine imported into the matrix by Hem25p can also be used for the synthesis of folate derivatives through the glycine cleavage system [108].

## 5. Future Perspectives

The transport properties of many MCs that transport different amino acids have been well characterized and shown to transport aspartate, glutamate, ornithine, citrulline, lysine, arginine, histidine and glycine with varying substrate specificity. Further investigation should be aimed at better exploring how the in vitro activities of characterized MCs for amino acids (some of which exist in multiple isoforms) are correlated with their suggested specific physiological roles in vivo, in various organisms, tissues and cell types. Moreover, future work is warranted to identify which of the remaining orphan MCs transport amino acids. Considering that identified human MCs that transport amino acids have R[DE] in contact point II (with the exception of ORC2 and UCP2 that have QE and RN, respectively), SLC25A39, SLC25A40, SLC25A45, SLC25A47 and SLC25A48 are likely candidates for amino acid transport. Among the amino acids that are expected to be transported into mitochondria are proline and branched-chain amino acids that are oxidized in mitochondria; however, until now no transporter has been identified for them or relatives of those amino acids. Additionally, the amino acids γ-aminobutyrate and δ-aminolevulinate are thought to be transported across the mitochondrial inner membrane, yet their transporters remain unknown. It could be, of course, that some amino acids are not transported by MCs but rather by mitochondrial transporters of other protein families. For example, SFXN1 has recently been shown to transport serine [109]. 

The MCs for amino acids that have been identified until now have roles in the bulk transport of amino acids that are metabolites in pathways or are recycled, such as in the malate-aspartate shuttle or urea cycle. In theory, the MCs for amino acids could also contribute to providing the building blocks for translating mitochondrial DNA-encoded proteins. This raises the question as to whether mitochondrial translation requires amino acid transport from the cytoplasm. Recent arguments against the necessity of amino acid transport across the mitochondrial inner membrane for mitochondrial protein synthesis have been based on the following points: (i) most of the characterized MCs for amino acids are strict antiporters, incapable of a net transport of amino acids with the exception of the GCs, SLC25A29 and GlyC, which could contribute to glutamate, arginine, lysine (histidine) and glycine import; (ii) almost all of the 35 MC family members in *S. cerevisiae* have been characterized, and it is unlikely that those remaining (YFR045wp and YGR257cp) transport all the other amino acids required for protein synthesis; (iii) the great majority of mitochondrial matrix proteins (99%) are imported [110], and the turnover of these proteins into single amino acids through degradation could provide sufficient amino acid concentrations for mitochondrial translation. Considering the third point, the initial question could be reformulated as follows: “Do mitochondria require amino acid export?” However, since all amino acids can be converted, at least indirectly, to TCA cycle metabolites, spare amino acids are probably oxidized and used in energy metabolism.

## Figures and Tables

**Table 1:** Sequence identities between the MC domains (of amino acid-transporting mitochondrial carriers) in percentages, with identities of 30% and above indicated in bold. The four main carrier groups are indicated with the same colors as in Figure 2.

	**AGC1**	**AGC2**	**Agc1p**	**GC1**	**GC2**	**AtUCP1**	**AtUCP2**	**UCP2**	**Ymc2p**	**BOU**	**ORC1**	**ORC2**	**Ort1p**	**BAC1**	**A29**	**BAC2**	**Hem25p**	**GlyC**
**AGC1**		**88**	**57**	**46**	**46**	28	28	27	24	**32**	27	27	27	25	**30**	**33**	21	25
**AGC2**	**88**		**58**	**46**	**46**	29	28	25	25	**31**	28	28	27	27	29	**33**	21	26
**Agc1p**	**57**	**58**		**42**	**39**	24	26	26	28	**32**	27	28	28	28	**33**	**30**	27	29
**GC1**	**46**	**46**	**42**		**68**	25	24	25	21	29	26	27	26	24	29	29	18	26
**GC2**	**46**	**46**	**39**	**68**		25	25	25	24	27	25	25	24	26	28	**31**	23	26
**AtUCP1**	28	29	24	25	25		**74**	**51**	26	**32**	24	22	24	24	**30**	27	19	23
**AtUCP2**	28	28	26	24	25	**74**		**45**	28	**30**	23	21	24	27	**31**	25	20	23
**UCP2**	27	25	26	25	25	**51**	**45**		25	29	25	25	25	24	27	27	20	25
**Ymc2p**	24	25	28	21	24	26	28	25		**41**	**31**	28	**30**	**31**	**39**	**30**	23	26
**BOU**	**32**	**31**	**32**	29	27	**32**	**30**	29	**41**		**40**	**38**	**33**	**36**	**38**	**36**	19	26
**ORC1**	27	28	27	26	25	24	23	25	**31**	**40**		**88**	**33**	**33**	**34**	**31**	24	25
**ORC2**	27	28	28	27	25	22	21	25	**28**	**38**	**88**		**31**	**34**	**34**	28	21	25
**Ort1p**	27	27	28	26	24	24	24	25	**30**	**33**	**33**	**31**		**31**	**31**	28	21	21
**BAC1**	25	27	28	24	26	24	27	24	**31**	**36**	**33**	**34**	**31**		**34**	29	20	26
**SLC25A29**	**30**	29	**33**	29	28	**30**	**31**	27	**39**	**38**	**34**	**34**	**31**	**34**		**40**	19	27
**BAC2**	**33**	**33**	**30**	29	**31**	27	25	27	**30**	**36**	**31**	28	28	29	**40**		26	26
**Hem25p**	21	21	27	18	23	19	20	20	23	19	24	21	21	20	19	26		**35**
**GlyC**	25	26	29	26	26	23	23	25	26	26	25	25	21	26	27	26	**35**	

## Note added during revision

During the revision of this paper a new report was published giving evidence that SLC25A44 transports branched-chain amino acids (Yoneshiro et al., (2019). BCAA catabolism in brown fat controls energy homeostasis through SLC25A44. Nature 572, 614-619).

## Abbreviations

AAC	ADP/ATP carrier, adenine nucleotide carrier or translocase
ADMA	asymmetric dimethylarginine
AGC	aspartate glutamate carrier
BAC	basic amino acid carrier
GC	glutamate carrier
EPRA	expression-purification-reconstitution-assay
MC	mitochondrial carrier
OGC	2-oxoglutarate carrier
ORC	ornithine carrier
TCA	tricarboxylic acid
UCP	uncoupling protein
